# Optimal caudal needle angulation for lumbar medial branch denervation: A 3D cadaveric and clinical imaging comparison study

**DOI:** 10.1016/j.inpm.2024.100433

**Published:** 2024-08-19

**Authors:** John Tran, Abdulrahman Alboog, Ujjoyinee Barua, Nicole Billias, Eldon Loh

**Affiliations:** a Division of Anatomy, Department of Surgery, University of Toronto, Toronto, Canada; b Department of Physical Medicine and Rehabilitation, Parkwood Institute, London, Canada; c Lawson Health Research Institute, London, Canada; d Department of Anesthesia and Perioperative Medicine, Western University, London, Canada; e Department of Anesthesia, Faculty of Medicine, University of Jeddah, Jeddah, Saudi Arabia

## Abstract

**Background:**

Lumbar medial branch (MB) radiofrequency ablation is a common intervention to treat facetogenic low back pain. The consensus among spine pain interventionalists is that capturing a greater length of the MB correlates with a longer duration of pain relief. Therefore, there has been interest in defining optimal needle angles to achieve parallel cannula placement. Presently, there is inconsistency regarding the optimal caudal needle angles.

**Objectives:**

The objectives of this study were to: 1) use a dissection-based 3D modelling methodology to quantify optimal caudal needle angles from cadaveric models; and 2) compare optimal cadaver-derived caudal needle angles with real-world patient-derived needle angles.

**Methods:**

Eighteen formalin embalmed lumbosacral spine specimens were dissected, digitized, and modelled in 3D. Virtual needles were simulated and placed parallel with the L1-L5 MBs. Cadaver-derived caudal needle angles were measured from the high-fidelity 3D models with optimally placed virtual needles. Lateral fluoroscopic images of patients (n = 200) that received lumbar MB denervation were reviewed to measure patient-derived caudal needle angles (L3-L5 MB levels). Descriptive statistics were used to analyze the cadaver (L1-L5 MB levels) and patient-derived (L3-L5 MB levels) caudal needle angles. The cadaver and patient-derived mean caudal needle angles for L3-L5 MB levels were compared.

**Results:**

There was variability in the cadaver-derived mean caudal needle angles. The lowest mean caudal needle angle was the L1 MB level measured at 41.57 ± 8.56° (range: 27.14° - 53.96°). The highest was the L5 MB level with a mean caudal needle angle of 60.79 ± 8.55° (range: 46.97° - 79.74°). A total of 123 patients were included and 369 caudal needle angles (L3-L5 MB levels) were measured and analyzed. There was variability in the patient-derived mean caudal needle angles. The patient-derived mean caudal needle angles were 29.18 ± 8.77° (range: 11.80° - 61.31°), 33.34 ± 7.23° (range: 16.40° - 54.15°), and 49.08 ± 8.87° (range: 26.45° - 76.95°) for the L3, L4, and L5 MB levels, respectively. There was a significant difference in mean caudal needle angle between cadaver and patient-derived needle angles at the L3, L4, and L5 MB levels.

**Conclusions:**

Analysis of cadaver-derived needle angles versus patient-derived data suggests optimization of lumbar MB denervation requires greater caudal angulation to achieve parallel needle placement. Further research is required to assess the clinical implications.

## 1 Introduction

Lumbar medial branch (MB) radiofrequency ablation (RFA) is a common intervention to treat facetogenic low back pain [1]. Traditionally, this procedure uses conventional needles with a parallel approach to ensure the entire length of the active tip is aligned with the MB to maximize nerve capture [2,4,5]. The consensus among spine pain interventionalists is that capturing a greater length of the MB correlates with a longer duration of pain relief [[2], [3], [4]]. Therefore, there has been interest in defining optimal needle angles to achieve parallel cannula placement with the MB [[5], [6], [7], [8], [9], [10]].

Previous studies investigating needle angles focused on using clinical imaging [7] or analysis of bony lumbar vertebrae [8] to define optimal degrees of angulation. However, these approaches do not directly measure angles based on the MB's course but use bony landmarks and prior knowledge of the nerve location. Consequently, there is inconsistency in the previous literature regarding the optimal caudal needle angles; clinical imaging and osteological studies have reported mean angle ranges of 20.15°–40.74° and 29.29°–47.22°, respectively [7,8]. Defining the optimal caudal needle angle to achieve parallel cannula placement is important as it can provide insights to improve technical performance of the procedure. Optimizing the parallel technique, from an anatomical perspective, will potentially translate into clinical outcomes that are more reflective of the approach's effectiveness to relieve facetogenic low back pain. This will enable more accurate comparison of pain relief outcomes between parallel and emerging nonparallel techniques to inform clinical practice. However, no previous studies were found that measured caudal needle angles based on the MB's course. This limits the ability to assess whether clinically used caudal needle angles are sufficient to achieve parallel placement.

With advances in 3D modelling technology, high-fidelity reconstruction of spatial relationships of nerve branches to bony landmarks from cadaveric specimens is now feasible [[11], [12], [13]]. Utilizing this technology to document the course of the MB and simulate parallel cannula placement will enable the accurate measurement of optimal caudal needle angles to assess clinical techniques. Therefore, the objectives of this study were to: 1) use a dissection-based 3D modelling methodology to quantify optimal caudal needle angles from cadaveric models; and 2) compare optimal cadaver-derived caudal needle angles with real-world patient-derived needle angles.

## 2 Materials and methods

### 2.1 Cadaver-derived data

#### 2.1.1 Cadaveric specimens, dissection, digitization, and 3D modelling

Eighteen formalin embalmed lumbosacral spine specimens with mean age of 75.4 ± 13.8 years (8 females/10 males) were used in this study. Specimens with signs of trauma or previous surgery were excluded. Ethics approval for this study was received from the University of Toronto Health Sciences Research Ethics Board (protocol #27210).

**Fig. 1:**
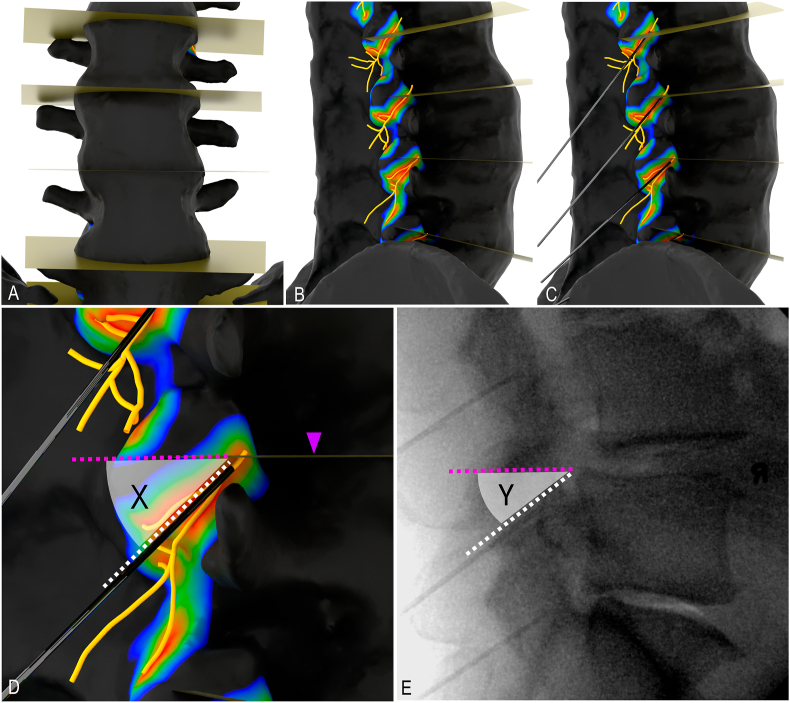
**Methodology.** A. Planes aligned parallel with superior vertebral endplates on high-fidelity 3D model reconstructed from cadaveric specimen, anterior view. B. Nerve proximity map based on 3D positional data of the lumbar medial branch, lateral view. C. Virtual needles placed with optimal caudal needle angles on high-fidelity 3D model, lateral view. D. Cadaver-derived caudal needle angle (X) measurement, lateral view. E. Patient-derived caudal needle angle (Y) measurement, lateral view. Purple arrowhead indicates plane aligned with squared superior vertebral endplate; purple dotted line, origin arm of angle; white dotted line, terminal arm aligned with needle. (For interpretation of the references to colour in this figure legend, the reader is referred to the Web version of this article.)

In each specimen, the L1-L5 dorsal rami were dissected, digitized and modelled in 3D (Fig. 1). The dissection, digitization and 3D modelling protocol has been previously described and published [13]. Eighteen high-fidelity 3D models of the lumbar dorsal rami and lumbosacral spines were reconstructed from the cadaveric specimens using Blender3D. The models documented the course of the branches of the lumbar dorsal rami in 3D space and relative to anatomical landmarks.

#### 2.1.2 Nerve proximity mapping, needle placement simulation, and cadaver-derived angle measurement protocol

The high-fidelity 3D models were used to generate nerve proximity maps for the L1-L5 MBs in each specimen (Fig. 1B). The nerve proximity mapping protocol has been previously described and published [14]. Virtual needles were simulated and placed along the periosteum of the lateral neck of the superior articular process (SAP) parallel with the L1-L5 MBs (Fig. 1C). The nerve proximity map provided a visual guide to ensure that each virtual needle was positioned optimally along the periosteum of the middle two quarters of the neck of SAP from a lateral view. Specifically, the caudal angulation of the virtual needle was finely adjusted so that the needle overlapped the red zone, within the nerve proximity map, indicative of the area where the MB is in contact with the periosteum [14]. Next, cadaver-derived caudal needle angles were measured from the high-fidelity 3D models with optimally placed virtual needles. To standardize the measurements, each caudal needle angle was measured with the corresponding superior vertebral endplate squared (i.e., direct lateral view). To acquire the direct lateral view, a transverse plane was modelled and aligned parallel to the superior vertebral endplate of the L1-L5 vertebrae and sacrum (Fig. 1A). By manipulating the high-fidelity 3D model, a direct lateral view was acquired when the plane (corresponding to the superior vertebral endplate) was visible as a straight line (Fig. 1 D). The 3D model was then rendered as an image and imported into ImageJ (opensource image processing software). Using ImageJ's built-in angle measurement tool, the caudal needle angle for each MB was quantified in a counterclockwise direction, where the line representing the posterior extension of the squared superior vertebral endplate was designated the initial arm and the virtual needle the terminal arm (Fig. 1D).

### 2.2 Patient-derived data

#### 2.2.1 Study design and setting

This was a single-center, retrospective study analyzing the electronic medical record (Powerchart) from St. Joseph Healthcare London (SJHC) Pain Clinic, a hospital-based tertiary pain clinic in London, Ontario, Canada. Lateral fluoroscopic images of patients that received lumbar MB denervation were reviewed to measure caudal needle angles. Patient-derived caudal needle angles were compared with cadaver-derived needle angles obtained from section 2.1. The Western University's Research Ethics Board approved this study (Protocol #120888).

#### 2.2.2 Selection criteria

This retrospective study included patients who had lateral fluoroscopic images captured during their treatment using the traditional parallel technique targeting the L3-L5 MBs between January 2017 and May 2022. Exclusion criteria included patients who had missing/inadequate lateral images (e.g. vertebral endplate at the level of interest was not square) or were treated with non-traditional denervation techniques (i.e. perpendicular/parasagittal approaches) for the L3-L5 MB levels.

#### 2.2.3 Intervention

All patients included in the current retrospective study received L3-L5 MB denervation using a traditional parallel technique with conventional radiofrequency cannulae. The procedure was carried out under fluoroscopic guidance according to published technical recommendations and guidelines for the traditional parallel technique that targets the middle two quarters of the lateral neck of SAP [2,4,5]. That is, during cannula placement using the traditional parallel technique, a caudal angulation of 20–40° from an image of the squared endplate at the level of interest was obtained. For the L3 and L4 MBs, approximately 20° obliquity was also incorporated [5]; L5 MB was often approached without any obliquity. Final angles for placement varied based on the ability of the interventionalist to visualize the intended target area at the junction of the SAP and the transverse process. The cannula was then placed using a “down the barrel” approach to the middle two-quarters of the SAP.

#### 2.2.4 Patient-derived needle angle measurement protocol

The electronic medical record (EMR) from SJHC Pain Clinic was queried for lateral fluoroscopic images with needle placement using the traditional lumbar MB denervation technique targeting L3-L5 MB levels. Caudal needle angles were measured from lateral fluoroscopic images using the angle measurement tool available on the EMR's imaging platform (Enterprise Imaging XERO Viewer, AGFA Healthcare; Mortsel, Belgium). Needle angle measurements were standardized by aligning the origin arm of the angle with the squared superior vertebral endplate and the terminal arm with the needle visible on the fluoroscopic image (Fig. 1E). Caudal needle angles were measured for the L3-L5 MB levels.

### 2.3 Data analysis

Descriptive statistics (means and ranges) were used to analyze the cadaver (L1-L5 MB levels) and patient-derived (L3-L5 MB levels) caudal needle angles. The cadaver and patient-derived mean caudal needle angles for L3-L5 MB levels were compared as these are commonly targeted clinically. Differences between cadaver and patient-derived mean caudal needle angles for each level (L3-L5) were analyzed using an independent-sample *t*-test to determine significance. Statistical analyses were performed using SPSS v28.0.1.0.

## 3 Results

### 3.1 Cadaver-derived mean caudal needle angles

**Fig. 2:**
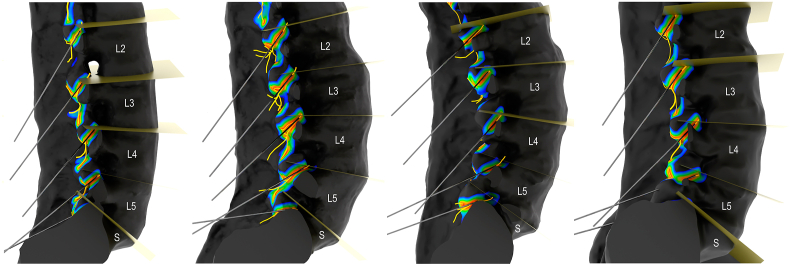
**High-fidelity 3D models reconstructed from four different cadaveric specimens, lateral views.** Virtual needles placed with optimal caudal angulation parallel along L1-L5 medial branches.

**Fig. 3:**
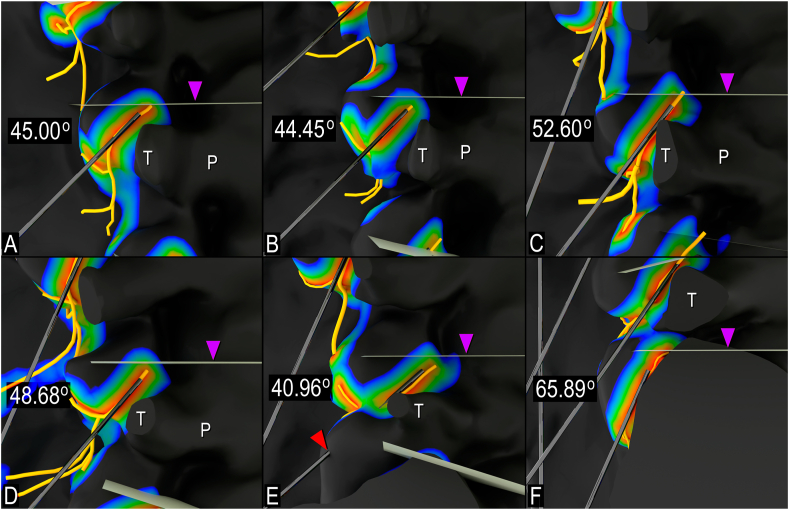
**Cadaver-derived caudal needle angles from high-fidelity 3D models to achieve parallel placement with medial branch (MB), lateral views.** A. L1 MB level. B. L2 MB level. C. L3 MB level. D. L4 MB level. E. L4 MB level with degenerative changes (red arrowhead) at L5/Sacral facet joint impeding needle trajectory. F. L5 MB level. Purple arrowhead indicates squared superior vertebral endplate; P, pedicle; T, transverse process. (For interpretation of the references to colour in this figure legend, the reader is referred to the Web version of this article.)

Ninety virtual needles were simulated and placed parallel to the L1-L5 MBs located along the lateral neck of SAP (Fig. 2, Fig. 3). For each MB level (L1-L5), 18 optimal caudal needle angles were quantified. There was variability in the cadaver-derived mean caudal needle angles for the L1-L5 MB levels (Table 1). The lowest mean caudal needle angle was the L1 MB level measured at 41.57 ± 8.56° (range: 27.14° - 53.96°). The highest was the L5 MB level with a mean caudal needle angle of 60.79 ± 8.55° (range: 46.97° - 79.74°). At the L4 MB level, 2 out of 18 cases (11.11 %) had degenerative/osteophyte formation at the L5-Sacrum facet joint which resulted in intersection with the optimally placed virtual needle due to the substantial degree of caudal angulation (Fig. 3E).

**Table 1:** Mean caudal needle angles quantified from cadaveric and patient data.

	Cadaver-derived data (n = 18)		Patient-derived Data (n = 123)		Mean difference (^o^)
	Mean caudal needle angle (^o^)	Range (^o^)	Mean caudal needle angle (^o^)	Range (^o^)	
L1	41.57 ± 8.56	27.14–53.96	n/a	n/a	n/a
L2	45.35 ± 10.35	20.33–66.72	n/a	n/a	n/a
L3	48.12 ± 6.18	36.30–57.52	29.18 ± 8.77	11.80–61.31	18.94
L4	48.40 ± 7.31	37.32–59.83	33.34 ± 7.23	16.40–54.15	15.06
L5	60.79 ± 8.55	46.97–79.74	49.08 ± 8.87	26.45–76.95	11.70

### 3.2 Patient-derived mean caudal needle angles

**Fig. 4:**
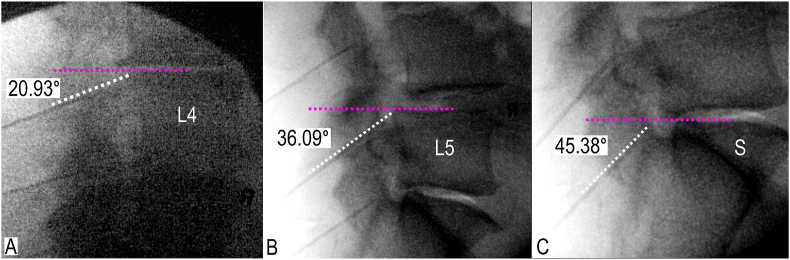
**Patient-derived caudal needle angles from fluoroscopic images, lateral views.** A. Caudal needle angle targeting L3 medial branch with fourth lumbar vertebra (L4) squared. B. Caudal needle angle targeting L4 medial branch with fifth lumbar vertebra (L5) squared. C. Caudal needle angle targeting L5 medial branch with sacrum (S) squared. Purple dotted line indicates the originate arm of angle aligned parallel with squared superior vertebral endplate; white dotted line, terminal arm of angle aligned with radiofrequency cannula. (For interpretation of the references to colour in this figure legend, the reader is referred to the Web version of this article.)

In total, 200 charts of patients who received lumbar MB denervation were reviewed, and 77 were excluded. Forty-one out of the 77 patients were excluded from the analysis for receiving treatment with non-traditional approaches. The remaining 36 patients were excluded for missing lateral radiographs i.e. lack of squared endplates at the levels of interest. A total of 123 patients were included and 369 caudal needle angles (123 patients x 3 MB levels) were measured and analyzed (Fig. 4). There was variability in the patient-derived mean caudal needle angles between the L3-L5 MB levels (Table 1). The patient-derived mean caudal needle angles were 29.18 ± 8.77° (range: 11.80° - 61.31°), 33.34 ± 7.23° (range: 16.40° - 54.15°), and 49.08 ± 8.87° (range: 26.45° - 76.95°) for the L3, L4, and L5 MB levels, respectively.

### 3.3 Cadaver vs. patient-derived mean caudal needle angles

There was a significant difference in mean caudal needle angle between cadaver and patient derived needle angles at the L3, L4, and L5 MB levels. At the L3 MB level, the cadaver-derived mean caudal needle angle was 18.94° greater than the patient-derived angle (*t*_139_ = 8.833, *p* < .001). At the L4 MB level, cadaver-derived mean caudal needle angle was 15.06° greater than the patient-derived angle (*t*_139_ = 8.245, *p* < .001). The cadaveric-derived mean caudal needle angle at the L5 MB level was 11.70° greater than the patient-derived angle (*t*_139_ = 5.249, *p* < .001).

## 4 Discussion

Robust anatomical knowledge is important to inform technical performance of lumbar MB denervation procedures. When using a parallel approach with a conventional cannula, appropriate angulation is essential to maximize needle-to-nerve contact to ensure optimal coagulation of the nerve target. The findings in the current study provide spine pain interventionalists with the knowledge needed to maximize needle-to-nerve contact, potentially improving pain relief outcomes.

### 4.1 Caudal needle angles

In previous literature, caudal angulations of needles have been investigated [[7], [8], [9]]. Patel et al. reported mean angle of insertion ranging from 20° - 40° caudally to the superior vertebral endplate and discussed their findings in the context of the previous recommendation of 35° - 40° caudal angulation [7]. More recent studies have reported the caudal needle angle to be greater than previous recommendations [8,9]. In the current study, using a dissection-based 3D modelling methodology, caudal needle angles were defined from cadaveric specimens. Specifically, caudal needle angles were measured based on the course of the lumbar MB. These values represent optimal caudal needle angles to inform parallel placement in the lateral view. The optimal cadaver-derived mean caudal need angles ranged from 41.57° - 60.79°, supporting the need for greater caudal angulation to achieve parallel placement with the lumbar MB as compared to previous recommendations [7]. The difference in previously recommended caudal needle angles may be attributed to angle measurements using different bony landmarks [7,8]. The present study supports the bony landmarks previous described which include the inferior margin of the mammillary process [6]. Moreover, in a recent case series it was reported that using a greater caudal angulation is effective [10]; however additional clinical investigation is required.

### 4.2 Technical optimization of lumbar medial branch denervation

Anatomical evidence is essential to assess and optimize image-guided RFA techniques. By comparing the optimal cadaver-derived caudal needle angles with patient-derived values, the finding in the present study allows for an evidence-based assessment of current approaches to achieve parallel placement with the lumbar MB. The mean caudal needle angles for the L3, L4, and L5 MB levels were found to differ between the cadaver and patient-derived data. Specifically, the mean cadaver-derived needle angles were greater by 18.94°, 15.06°, and 11.70° for the L3, L4, and L5 MB levels, respectively. This suggests that the parallel technique, as currently performed at this academic institution, does not achieve sufficient caudal angulation in needle placement to maximize parallel alignment along the MB which corroborates with previously published literature [6].

As non-parallel approaches continue to be developed (e.g. expanded lesion/multi-tined cannulae) and compared to the parallel approach, it is important to ensure that the parallel approach is performed with sufficient caudal angulation. This is important to determine the true clinical effectiveness of the parallel approach. Consideration of differences in pain relief, procedure time, and equipment cost of the parallel technique (using a conventional needle) vs a non-parallel approach (with expanded lesion/multi-tined cannula) is necessary and requires further clinical investigation.

Needle angles to achieve parallel placement, in the posterior view, have also been discussed and recently investigated [[7], [8], [9]]. Depending on the patient's anatomy and the preference of the interventionalist, targeting different portions of the lateral neck requires the needle to have different degrees of obliquity. Generally, when targeting the middle two quarters of the lateral neck, an oblique angulation from the sagittal plane is necessary [8], whereas targeting the posterior half requires parasagittal angulation [9]. Regardless of the degree of needle obliquity on the posterior view, which would impact the depth the needle needs to be advanced along the lateral neck of SAP (i.e. greater obliquity requires deeper placement), the result of the current study suggests greater caudal angulation than previously recommended (20–40°) [7] is necessary to achieve parallel alignment with the lumbar MB. Greater caudal angulation can be achieved if the mammillary process is visualized on the lateral view, and the cannula placed between the mammillary process and the accessory process within the mamilloaccessory notch, as described in previous anatomical studies [6,8].

### 4.3 Patient-specific needle angles

The findings in the current study suggest optimization of lumber MB requires greater caudal angulation. However, based on the range of optimal caudal needle angles, as reported in the current study, a patient-specific approach using landmarks rather than average needle angle may be more appropriate to optimize patient outcomes. Furthermore, the presence of osteophytes and degenerative changes may represent physical barriers to achieving optimal parallel placement. In the current study 11.11 % of needles targeting the L4 MB level were occluded by an osteophyte at the L5/sacrum facet joint due to the substantial degree of caudal angulation required to achieve parallel placement with the nerve. Continued anatomical research is needed to correlate 3D anatomy with detailed 2D fluoroscopic features to develop a guide for spine pain interventionalists in selecting the most optimal needle placement approach based on patient-specific anatomy.

### 4.4 Limitations

The current study is limited by a small sample size due to the laborious nature of anatomical research. Consequently, the documented anatomy does not encompass all potential interpersonal differences and degenerative changes. However, the current study's sample size is greater than the recommended number for studies with no previous data [15]. Another limitation is that the patient-derived caudal needle angles for this study were obtained from only one academic institution. As such, any differences in lumbar RFA technique at other institutions/clinics may not be reflected in the current study. As an anatomy comparison study, all implications discussed require further investigation.

## 5 Conclusions

In this 3D anatomical-clinical imaging comparison study, caudal needle angles were quantified and compared. Analysis of cadaver-derived needle angles versus patient-derived data suggests optimization of lumbar MB denervation requires greater caudal angulation to achieve parallel needle placement as compared to previous recommendation [7]. Greater caudal angulation can be achieved if the mammillary process is visualized on the lateral view, and the cannula placed between the mammillary process and the accessory process within the mamilloaccessory notch, as described in a previous anatomic study [6]. The angulation necessary to achieve this is greater than the 20–40° caudal angulation previously recommended in the literature [7]. The range of optimal cadaver-derived caudal needle angles reflects the anatomical variations that are present between individuals; clearly defined criteria for patient-specific approaches (based on robust anatomical landmarks) [6] may therefore be necessary to inform needle placement. The current study adds to the available literature by 1) quantifying optimal caudal needle angles by simulating cannula placement parallel with the MB reconstructed from cadaveric dissection and 2) provide further evidence corroborating the previously identified bony landmarks to achieve parallel alignment with the MB [6,8].”

## Declaration of competing interest

The authors declare the following financial interests/personal relationships which may be considered as potential competing interests:

Study was funded by IPSIS research grant awarded to Drs Eldon Loh (PI) and John Tran (Co-I) If there are other authors, they declare that they have no known competing financial interests or personal relationships that could have appeared to influence the work reported in this paper.
